# An Approach to Control Relapse of Inflammatory Lesions after Discontinuation of Primary Therapy

**DOI:** 10.1371/journal.pone.0098051

**Published:** 2014-05-20

**Authors:** Pradeep B. J. Reddy, Sharvan Sehrawat, Amol Suryawanshi, Naveen K. Rajasagi, Madhu Khatri, Barry T. Rouse

**Affiliations:** Department of Pathobiology, College of Veterinary Medicine, University of Tennessee, Knoxville, Tennessee, United States of America; University of Georgia, United States of America

## Abstract

Long-term treatment with the fungal metabolite drug FTY720 (Fingolimod) was shown to be highly effective in controlling viral immunopathological lesions. However, in this report we show that the anti-inflammatory effect of FTY720 in herpes simplex virus-1 (HSV-1) induced ocular inflammation is lost upon the discontinuation of treatment and lesions rapidly recurred. The lesions that developed after FTY720 treatment withdrawal involved mainly Th17 cells rather than Th1 cells explained in part by differential expression of surface CD103, an integrin that permits migration of effector cells to inflammatory sites. The expression of IL-6, a proinflammatory cytokine involved in the generation of Th17 cells, was found to be increased in FTY treated mice as compared to controls and this effect could be abrogated upon administration of neutralizing antibody to IL-6. Furthermore, IL-17RKO mice failed to show the recurrence of stromal keratitis (SK) lesions upon FTY720 withdrawal. These results indicate that approaches such as neutralization of proinflammatory cytokines might be considered along with FTY720 treatment if interruption of drug therapy becomes necessary.

## Introduction

Chronic lesions of stromal keratitis (SK) in the eye that occur in response to HSV-1 infection are mainly orchestrated by CD4^+^ T cells, although target antigens that drives the inflammatory process have yet to be identified [1]. Control of lesion expression can be accomplished in several ways. These include inhibiting the effector function of the T cell orchestrators [2] and expansion of Foxp3 regulatory T cells [3], [4]. Changes in balance between effector T cells and Tregs occur when infected animals are continuously treated with the fungal metabolite drug FTY720 [5]. This drug binds to sphingosine 1 phosphate (S1P) receptors and modulates their surface expression thereby rendering them unresponsive to S1P in the blood plasma [6], [7], [8]. One outcome of such an effect is the retention of lymphocytes in lymphoid organs and their limited access to inflammatory sites [9], [10]. An additional means by which FTY720 succeeds in controlling inflammation is by inducing the conversion of conventional T cells into Foxp3^+^ regulatory T cells [5], [11]. The lymphocyte sequestration effect can be reversible and cells can repopulate the periphery upon treatment withdrawal [12]. This effect could be potentially damaging if FTY720 is used to treat chronic inflammatory conditions where stimulating insults, such as by antigens or after stimulants of the inflammatory milieu persists [13].

In the present report, we note that discontinuation of FTY720 at 10 days post infection (pi.) a time when virus is usually cleared from corneas of untreated animals with SK, resulted in a rebound of SK lesion severity to reach, or even exceed, that noted in untreated animals. This rebound was attributed to a reinvasion of the cornea with mainly Th17 effector T cells rather than Th1 cells as normally dominate SK lesions [14], [15]. The cause for the rebound could not be fully defined, but neutralization of the cytokine IL-6 which is involved in Th17 generation prevented or markedly suppressed the rebound effect and the effect was barely evident in IL-17 receptor KO mice when FTY720 treatment was withdrawn. Our results indicate that relapse of chronic inflammatory lesions and possibly autoimmune diseases can be prevented by using combination therapy with drugs such as FTY720 along with neutralization of proinflammatory cytokines required for the generation of Th17 cells.

## Materials and Methods

### Mice

Female 6 to 8 week old C57BL/6 mice were purchased from Harlan Sprague Dawley Laboratory (Indianapolis, IN). IL-17RKO mice were obtained from Amgen (Thousand Oaks, CA). DO11.10 RAG2−/− mice were obtained from Taconic farm. Animals were housed in the animal facilities approved by the Association for Assessment and Accreditation of Laboratory Animal Care at the University of Tennessee.

### Ethics Statement

This study was carried out in strict accordance with the recommendations in the Guide for the Care and Use of Laboratory Animals of the National Institutes of Health. The protocol was approved by the University of Tennessee Animal Care and Use committee (protocol approval numbers 1253-0412 and 1244-0412). All procedures were performed under tribromoethanol (avertin) anesthesia, and all efforts were made to minimize suffering.

### Virus and Reagents

HSV-1 RE was propagated and titrated on Vero cells (American Type Culture Collection CCL81) using standard protocols. The virus was stored in aliquots at −80°C until use. All antibodies were purchased from BD Pharmingen unless otherwise stated. The Abs used for flow cytometry were CD4-APC (RM4–5), CD25-FITC (7D4), Foxp3-PE (FJK-16s), CD62L-FITC (MEL-14), CD103-FITC (M290), CD45-APC (30-F11). Anti-CD3 and anti-CD28 (37.51) were from BD Biosciences. rhIL-2 was obtained from Peprotech and FTY720 from Cayman Chemicals. FTY720 was dissolved in ethanol at a concentration of 10 mg/ml, and before injecting mice, a fresh solution was made in distilled water. OVA_323–339_ peptide was obtained from Gen Script. CFSE was purchased from Molecular probes and used at a final concentration of 0.5 µM for 15 min at 37°C in PBS.

### Corneal HSV-1 Infections, Clinical Observations, and Treatment of Mice with FTY720 and anti-IL-6 Antibody

Mice were ocularly infected with 1×10^4^ PFU of HSV and divided randomly into four groups. Animals were treated with various doses (0.03, 0.3 and 3.0 mg/kg body weight (BW)) of FTY720 i.p. daily starting from 24 hrs pi. until either day 10 or day 14 pi. as described previously [5]. Mice were observed for the development and progression of herpetic stromal keratitis (SK) lesions and angiogenesis from day 5 until day 15, as described elsewhere [3]. The eyes were examined on different days pi. and the SK lesion severity and angiogenesis of individual mice were recorded. In few experiments C57BL/6 mice infected with 1×10^4^ PFU of HSV-1 were left untreated which served as controls. Second group of mice received FTY720 starting from 24 hrs pi. until 14 days pi. Third group of mice received FTY720 until day 10 pi. and later FTY720 treatment was discontinued. Fourth group of mice received FTY720 until day 10 pi and this group was also treated with IL-6 neutralizing antibody every alternate day starting from day 1 pi. until day 14 pi. Fifth group of mice received only IL-6 neutralizing antibody treatment every alternate day until the day 14 pi. and the disease progression was observed. All these experiments were repeated at least three times.

### Differentiation of Naïve CD4^+^ T Cells in to Th1, Th17 and Regulatory T Cell Subsets

To measure the effect of FTY720 treatment on differentiation of CD4^+^ T cells into Th1, Th17 and regulatory T cell subtypes, DO11.10 RAG2−/− mice were treated with 0.3 mg/kg of FTY720 for seven days. Splenocytes from control and treated mice were isolated and differentiation of naïve CD4^+^ T cells into Th1 (anti-CD3 5 µg/ml anti-CD28 1 µg/ml, IL-2 20 ng/ml, IL-12 20 ng/ml, anti-IL-4 10 µg/ml); Th17 (anti-CD3 5 µg/ml anti-CD28 1 µg/ml, TGF-β 1 ng/ml, IL-6 100 ng/ml, anti-IL-4 10 µg/ml, anti-IFN-γ 10 µg/ml) and Tregs was performed essentially following a method described elsewhere [4], [16], [17]. Th1 and Treg cell differentiation was performed in RPMI medium where as Th17 differentiation was performed using IMDM medium. After 5 days of incubation, Th1 and Th17 polarized cells were stimulated with PMA/Ionomycin in the presence of Brefeldin A for 4 h and stained for IFN-γ and IL-17 producing cells by intracellular staining (ICCS). Naïve CD4^+^ T cells polarized under Treg differentiating conditions were stained for Foxp3 transcription factor.

### Flow Cytometry

In vitro cultured cells, peripheral blood cells or cells isolated from lymphoid organs were surface stained with fluorochrome-labeled antibodies. For Foxp3 staining, a kit from eBioscience was used. Cells were isolated from corneal tissues by digesting with 60 U/ml liberase (Roche Diagnostics) for 45 min at 37°C in a humidified atmosphere of 5% CO_2_ as described earlier [18]. After incubation, the corneas were disrupted by grinding with a syringe plunger on a cell strainer, and single-cell suspensions were made in complete RPMI 1640 medium. The single-cell suspensions obtained from the corneas, draining lymph nodes (DLNs), and spleens were stained for different cell surface molecules for FACS. Cell suspensions were blocked with an unconjugated anti-CD32/CD16 mAb for 30 min in FACS buffer. After being washed with FACS buffer, samples were incubated with CD4-APC for 30 min on ice. Finally, the cells were washed three times and resuspended in 1% paraformaldehyde.

To measure the number of IFN-γ and IL-17 producing CD4^+^ T cells, ICCS was performed. Briefly, 10^6^ freshly isolated cells from the corneas, DLNs, or spleens were left untreated or stimulated with PMA plus ionomycin along with Golgi Stop for 4 h at 37°C in 5% CO_2._ At the end of the stimulation period, cell surface staining was performed as described above, followed by intracellular cytokine staining using the BD Cytofix/Cytoperm kit (BD Pharmingen) in accordance with the manufacturer’s recommendations. FITC-labeled IFN-γ and PE-labeled IL-17 Abs were used. After the final wash, cells were resuspended in 1% paraformaldehyde. The stained samples were acquired with a FACS Calibur (BD Biosciences), and the data were analyzed using FlowJo software (Tree Star, Ashland, OR).

### ELISA

The levels of IL-6 were measured in the corneal homogenates by using kits from R and D as per the manufacturers instruction as mentioned previously [18]. Briefly, the corneal samples were pooled group wise (six corneas/group) triturated and homogenized using a tissue homogenizer (Pellet Pestle mortar; Kontes). The concentration of IL-6 cytokine was measured by sandwich ELISA.

### Statistical Analysis

Student t test and one-way ANOVA were used to calculate the levels of statistical significance between groups and the p values represented as mean ± SEM. *p≤0.05, **p≤0.01, and ***p≤0.001.

## Results

### Interruption of FTY720 Therapy Leads to Recurrence of SK Lesions

**Figure 1:**
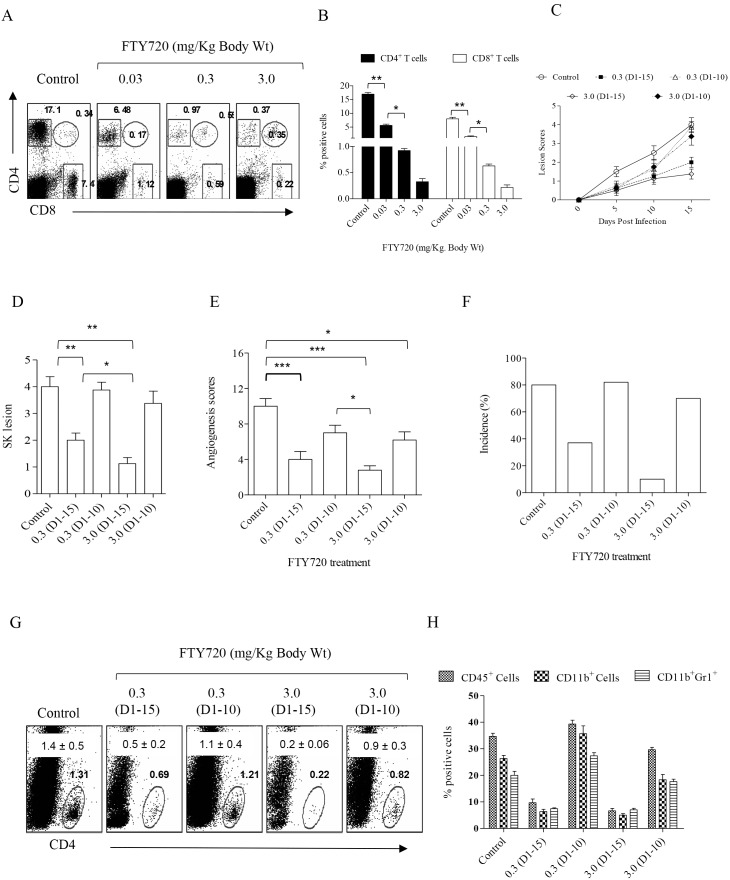
Continued therapy with FTY720 reduces the severity of SK lesions while discontinued treatment leads to relapse. C57BL/6 mice infected with 1×10^4^ PFU of HSV-1 were treated with FTY720 with various concentrations (0.03, 0.3 and 3.0 mg/Kg body weight) starting from 24 h pi. (A–B) FTY720 treatment depletes T cells from the periphery. (A) FACS plots showing the frequencies of CD4^+^ and CD8^+^ T cells from peripheral blood of FTY720 treated and control mice gated on total live cell population. (B) Bar graphs represents the frequencies of CD4^+^ and CD8^+^ T cells in blood isolated from FTY720 treated and control mice. (C) SK disease progression was measured in FTY720 and control groups at different time points till day 15 pi. (n = 5 to 6 mice per group). D–E. SK lesion (D) and angiogenesis (E) scores in different groups of mice on 15 dpi is shown. One-way ANOVA with Bonferroni’s multiple comparison test was used to calculate significance between control and FTY720 treated groups. (F) Bar graph depicts disease incidence (SK score >2.0) in various groups of mice measured on day 15 pi. Data represents mean ± SEM of at least two independent experiments. (G) FACS plots represent the frequencies of CD4^+^ T cell infiltration in various groups of mice. Numbers in the plots represent the cumulative data with SEM in indicated groups of mice. (H) Bar graphs showing the number of CD45^+^ CD11b^+^ double positive cells (macrophages/monocytes/dendritic cells) and CD45^+^CD11b^+^Gr1^+^ neutrophils in the corneas of different groups. Data represent means ± SEMs of at least two independent experiments. Statistical significance was calculated by one-way ANOVA (*p≤0.05, **p≤0.01, and ***p≤0.001).

Initial experiments involved FTY720 treatment beginning 24 hrs post HSV-1 infection and continued until day 15 pi. This procedure caused effective depletion of CD4^+^ as well as CD8^+^ T cells from the periphery (Figure 1A–B). This was accompanied by a delayed onset as well as overall suppression of ocular SK lesions and angiogenic responses in the ocular tissues of FTY720 treated mice (Figure 1C–F). The numbers and frequencies of the total CD4^+^ T cell population and other inflammatory cells that infiltrate the corneas of treated animals were dramatically reduced as compared to those in the control group (Figure 1G and H).

Because FTY720 is known to reversibly block the migration of inflammatory cells to the site of infection [6], we tested whether discontinuing FTY720 treatment at 10 days pi. a time when replicating virus is no longer detectable in untreated animals, would influence the beneficial effects of FTY720 treatment. In such experiments, lesions in FTY720 treated animals were barely evident but were readily apparent in untreated animals. It was evident that treatment withdrawal at 10 days pi. resulted in the rapid reappearance of lesions with them being of similar or even greater magnitude compared to untreated animals 5 days later (day 15 pi.). SK lesions on day 15 pi. in continually treated animals were minimal although still evident (Figure 1C–F).

At the termination of experiments, ocular tissues and DLNs were collected to quantify the presence of various cell types in the different groups. Abundance of CD4^+^ T cell subsets as well as innate immune cells such as neutrophils (CD45^+^CD11b^+^Gr1^+^) and macrophages/monocytes/dendritic cells as defined by CD45^+^CD11b^+^ expression were present in the corneas isolated from untreated controls on day 15 pi. (Figure 1G and H). In contrast, the responses in the continually treated group were minimal (Figure 1G and H). Similarly, minimal inflammatory cell infiltration as defined by CD45^+^ cells was observed in mice that were treated with FTY720 for 10 days and corneas analyzed on day 11 pi. Of the total live cells in the control and treated groups, 29±5.6% and 4.3±1.7% respectively were CD45^+^. However, corneal cellular preparations of animals from the treatment discontinuation group showed abundant inflammatory cell infiltrates by day 15 pi. (Figure 1G and H). In fact the total CD45^+^ cell numbers were comparable to those in the untreated group (∼35.6%±4% in controls vs 33.1%±7% in discontinued group).

**Figure 2:**
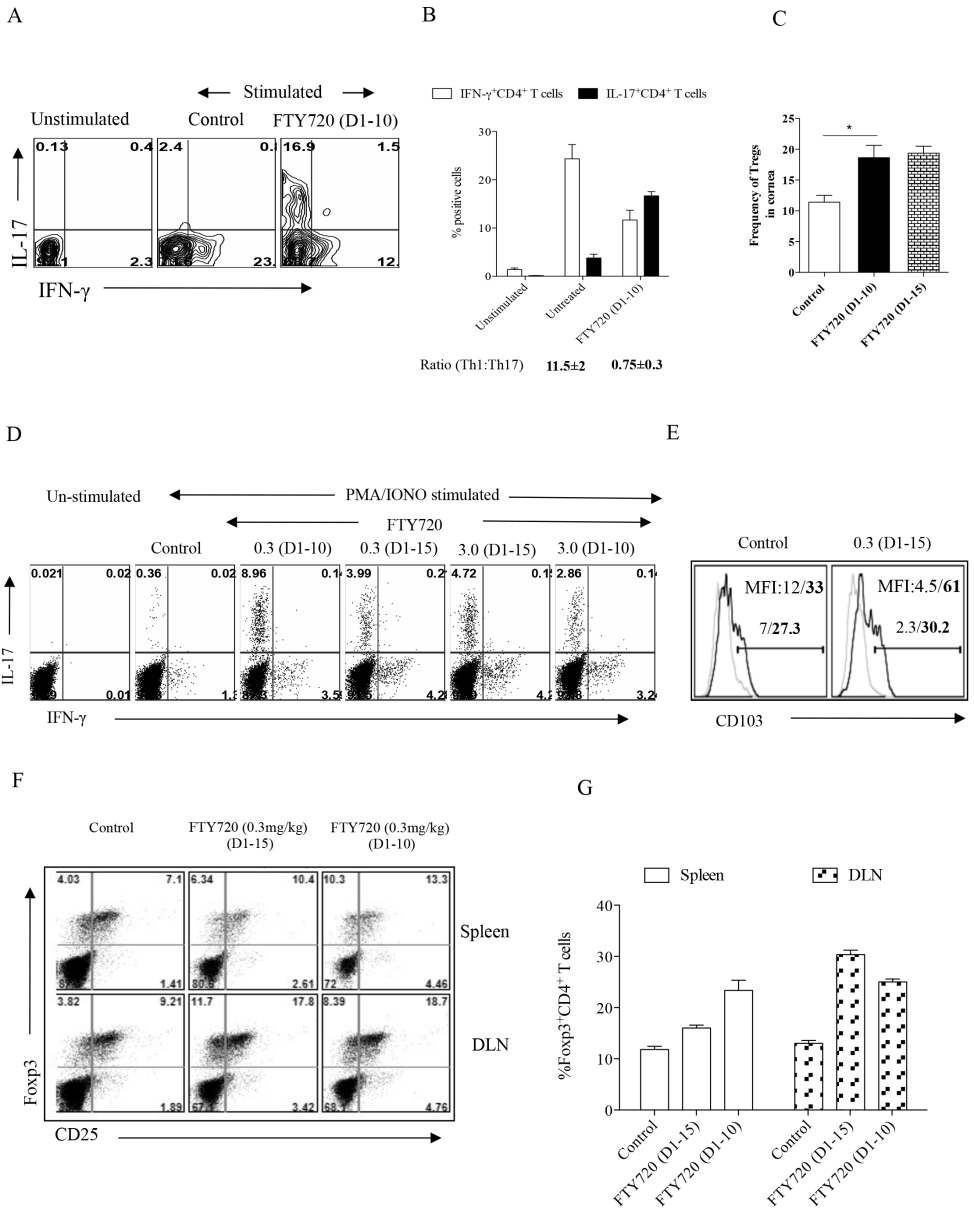
Continued therapy with FTY720 reduces proinflammatory cell infiltration while discontinued treatment leads to rapid influx of such cells. C57BL/6 mice infected with HSV-1 were treated with various concentrations of FTY720 or vehicle 24 h pi. Few groups of mice received FTY720 till day 15 pi and in few groups FTY720 treatment was discontinued by day 10 pi. HSV-1 infected corneas harvested on day 15 pi, were processed and stained for different cell surface and intracellular molecules. (A) FACS profiles showing the frequencies of CD4^+^ T cells isolated from cornea of FTY720 treated and control groups of mice that produce IFN-γ or IL-17 upon PMA/Ionomycin stimulation. (B) Bar graph represents the percentage of CD4^+^ T cells isolated from cornea that produce IFN-γ or IL-17 in various groups of mice. (C) Bar graph represents the frequencies of CD4^+^ Foxp3^+^ regulatory T cells present in corneas isolated from various groups. Data represents means ± SEM of at least two independent experiments. Statistical significance was calculated by one-way ANOVA with Tukey’s multiple comparison tests. (*p≤0.05). (D) FACS plots showing the frequencies of CD4^+^ T cells isolated from draining lymph nodes that produce IFN-γ and IL-17 upon stimulating with PMA/Ionomycin. (E) FACS plots showing CD103 expression on IFN-γ^+^ cells (thin lines) and IL-17^+^ cells (thick lines). Numbers in histograms represent % positive cell of IFN-γ^+^ cells (normal letter) and IL-17^+^ CD4^+^ cells (bold letter). Experiments were repeated 3 times with 5 to 6 mice per group. (F) FACS plots showing the frequencies of CD4^+^CD25^+^Foxp3^+^ T cells in the lymphoid organs (Upper panel-spleen, Lower panel-DLN) of mice treated with FTY720 (0.3 mg/Kg Body Wt) from day 1 until day 14 pi. and those that were discontinued from the therapy. (G) Bar graphs depict the percentage of CD4^+^CD25^+^Foxp3^+^ T cells isolated from lymphoid organs of FTY720 treated and control groups.

In vitro stimulation of pooled corneal inflammatory cells with PMA/Ionomycin revealed that whereas cells from the untreated animals revealed more abundant CD4^+^ T cells that were IFN-γ producers than IL-17 producers (Figure 2A and B), in the samples from the treatment withdrawal group the predominant CD4^+^ T cell type was IL-17 rather than IFN-γ producers (Figure 2A and B), skewing the ratio in favor of Th17 cells (Figure 2B). A similar pattern of results was noted with DLN cells with CD4^+^ IFN-γ^+^ cells outnumbering CD4^+^ Th17 cells in untreated but a reverse pattern was evident in the treatment withdrawal group (Figure 2D). These data infer that in untreated mice corneal samples CD4^+^ T cells were predominantly Th1 with minor numbers of Th17, but in the FTY720 treatment withdrawal group the majority of T cells were of the Th17 subtype.

On day 15 pi. the frequencies of Foxp3^+^ regulatory T cells in different groups was analyzed both in the cornea and DLNs. The data revealed that the frequencies of Tregs in corneas was approximately 1.6 fold higher in the FTY720 discontinued group compared to the control group (Figure 2C). Similarly the Tregs in DLNs and spleens of FTY720 treated group was almost 2 fold higher compared to the control group (Figure 2F and G).

Further experiments were performed with Th1 and Th17 cells from untreated and treatment discontinuation DLN population to measure and compare the expression of some key molecules involved in cell migration. While Th1 and Th17 cells both expressed similar levels of CD49d, a trend was observed in the numbers of Th17 cells that expressed CD103 to a greater extent as measured by mean fluorescence intensity (Figure 2E and data not shown). CD103 integrin is involved in the migration of cells to tissue sites and up regulation of this molecule on Th17 cells might be responsible for the preferential migration of these cells to corneal sites. However, there might be other factors that are involved in the preferential migration of such cells to inflammatory sites that we have not looked into.

### FTY720 Treatment Delays HSV Clearance from Infected Corneas

**Figure 3:**
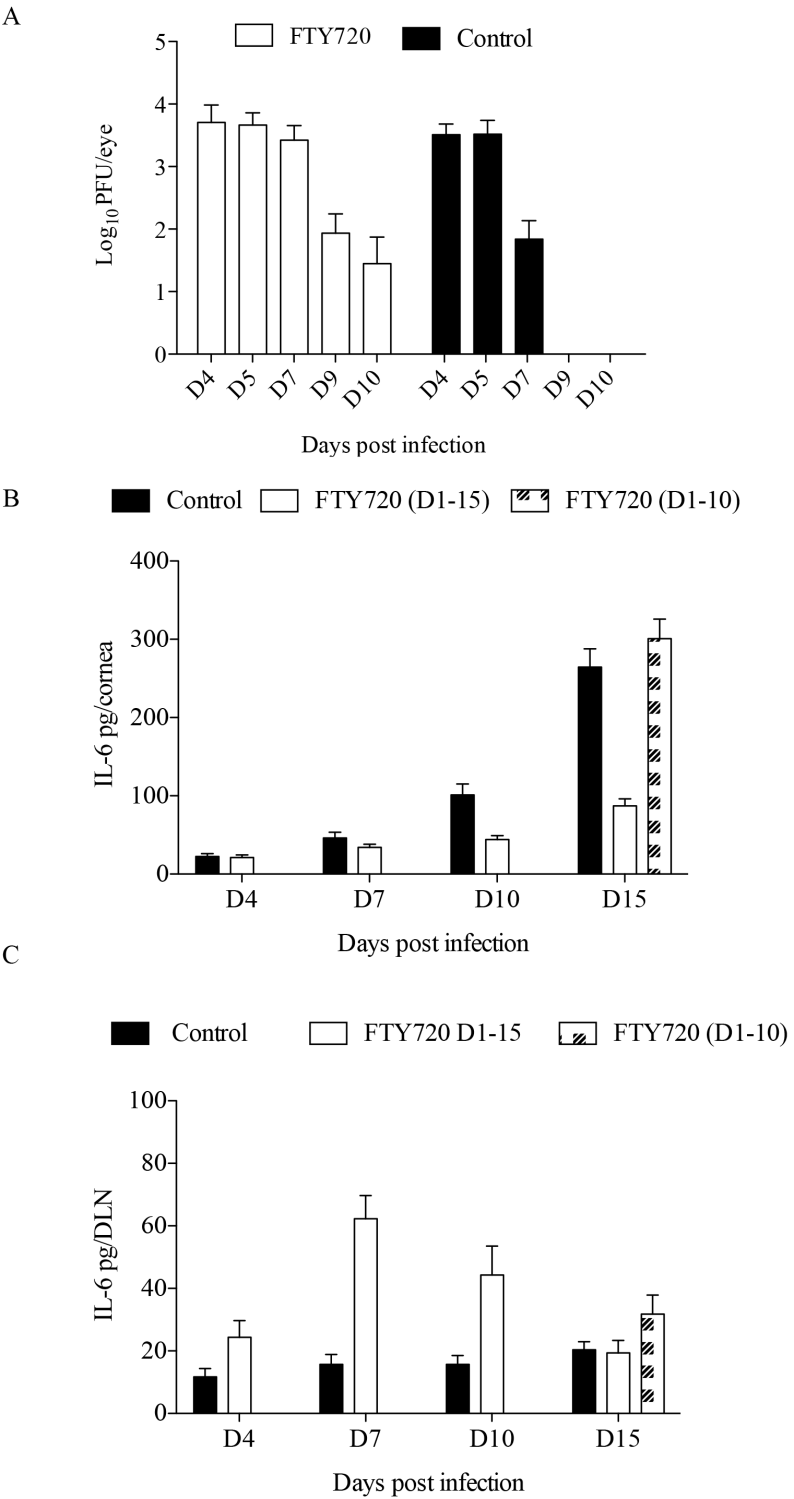
FTY720 treatment delays viral clearance from infected corneas and enhance IL-6 expression. C57BL/6 mice were infected with 1×10^4^ PFU of HSV-1. One group served as infected control and the other was treated with FTY720 (0.3 mg/kg body weight) starting from 24 h pi. (A) Corneal swabs were collected from treated and control groups at various days pi. and virus titers were determined using standard plaque assays using Vero cells. 4–5 mice per group for each time point was used to quantify the viral titers. (B) IL-6 concentration measured by sandwich ELISA in the corneal homogenate samples of FTY720 treated and control groups at different time points are shown. Pooled corneas from each group were used for the ELISA quantification. Bar graphs represent the levels of IL-6 in corneal samples of different groups of mice. Statistical levels of significance were analyzed by a Student t test. Data represent means ± SEM (C). Bar graphs represent the levels of IL-6 in DLNs of different groups of mice. Statistical levels of significance were analyzed by a Student t test. Data represent means ± SEM.

A rapid influx of CD4^+^ T cells was observed in the corneas of animals on the treatment discontinuation regimen. A possible explanation for the unexpected observation was that FTY720 treatment prevented the exit of T cells from lymphoid organs and this could result in delayed viral clearance from infected corneas. Support for this possibility was shown in experiments that quantified virus levels in the untreated and FTY720 treated groups. As shown in figure 3A, the viral titers were approximately *2 logs* higher in the corneas of FTY720 treated mice as compared to untreated mice at 7 days pi. Moreover, at 10 days pi. whereas replicating virus could no longer be detected in the corneas isolated from the untreated control group, low levels of infectious virus was still present in mice treated with FTY720. By 15 dpi, the virus was cleared from both the groups (data not shown). This finding could mean that the persisting virus might lead to remnant viral antigens that could provide a stimulus to drive the rebound of inflammatory immune cells once FTY720 treatment was withdrawn at 10 days pi.

### Neutralization of IL-6 Prevent the Recurrence of Lesions

The observation that lesion rebound occurred following discontinuation of FTY720 treatment could be explained by differential expression of cytokines/chemokines perhaps related in turn to the differences noted in viral persistence in treated vs untreated mice. Since the T cell type that dominated the rebound was Th17 cells a likely participant could be IL-6 known to be involved in Th17 cell differentiation [19], [20]. Moreover IL-6 is a predominant cytokine induced by HSV-1 infected cells and plays a critical role in SK pathogenesis [21], [22], [23], [24]. To assess a possible role of differential IL-6 expression, levels of this cytokine were compared at various time points in the corneas and DLNs of the different treatment groups by sandwich ELISA. A gradual increase in IL-6 levels was observed from day 4 until day 15 pi. in the untreated group with the IL-6 concentration 3 fold higher (264±23 pg/Cornea) in the corneas compared to the continuous treatment group (82±9 pg/Cornea) as measured on day 15 pi. (Figure 3B). However, IL-6 concentration in FTY720 discontinued group was 3 fold (300±25 pg/Cornea) higher than the group that received continuous treatment. This could be because of the influx of inflammatory cells in the corneas of mice discontinued with FTY720 therapy. Similarly, the DLN homogenates that were isolated from FTY720 treated mice on day 10 pi. exhibited 2.5 fold higher levels of IL-6 (43±11 pg/DLN) compared to those from untreated samples (15±7 pg/DLN) (Figure 3C). On day 15 pi. the IL-6 concentration was slightly higher in the FTY720 discontinued group (37±7 pg/DLN) compared to the other two groups. Collectively, these data indicate that the increased levels of IL-6 in FTY720 treated groups could be partly responsible for the polarization of Th17 cells.

**Figure 4:**
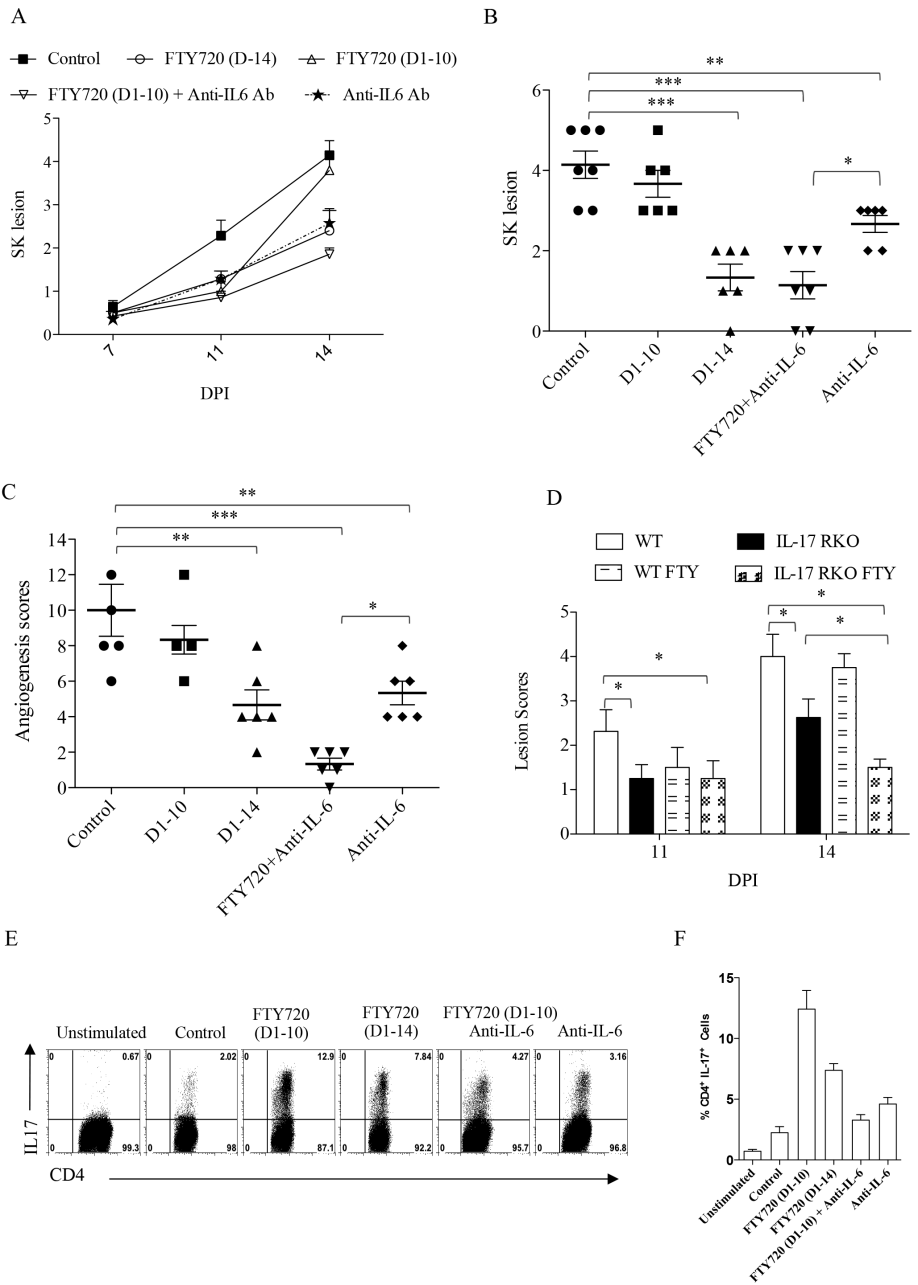
Inhibition of Th17 generation prevents recurrence of lesions in HSV-1 infected mice. C57BL/6 and IL-17 RKO mice were infected with 1×10^4^ PFU of HSV-1. First group of mice were left untreated which served as controls. Second group of mice received FTY720 starting from 24 hrs pi. until 14 days pi. Third group of mice received FTY720 until day 10 pi. and later FTY720 treatment was discontinued. Fourth group of mice received FTY720 until day 10 pi and this group was also treated with IL-6 neutralizing antibody every alternate day until the day 14 pi. Fifth group of mice received only IL-6 neutralizing antibody treatment every alternate day until the day 14 pi. and the disease progression was observed. (A) SK progression was measured in treated and control groups at different time points till day 15 pi. (B) SK lesion scores on day 15 pi. Statistical significance was calculated by one-way ANOVA (*p≤0.05, ** p≤0.01, and ***p≤0.001). (C) Angiogenesis scores were measured in anti-IL-6 Ab treated and control groups on day 15 pi. (n = 5 to 6 mice per group). (D) The comparative analysis of disease progression in WT and IL-17 RKO mice that were given FTY720 treatment or where treatment was withdrawn. SK lesion scores were measured on day 11 and 14 pi. in different groups of mice i.e., infected controls, infected control mice treated with FTY720 (0.3 mg/kg body wt) from day 1–10, IL-17RKO mice and IL-17 RKO mice treated from day 1–10 with FTY720 (0.3 mg/kg body wt) from day 1–10 pi. Per group five mice were used and the experiments were repeated twice. Statistical significance was calculated by one-way ANOVA (*p≤0.05, **p≤0.01, and ***p≤0.001). (E) FACS plots representing the frequencies of IL-17 producing cells isolated from draining lymph nodes of various groups and stimulated in the presence of PMA/Ionomycin. (F) Cumulative data for the production of Th17 in different groups of mice is represented by bar graphs.

Further evidence for a role for IL-6 to account for lesion rebound came from experiments using neutralization antibody to IL-6. Mice infected with HSV-1 were treated with IL-6 neutralizing antibody every alternate day starting from 24 hr pi. until the day of termination of experiments and lesion development was scored. As shown in Figure 4A–C, IL-6 neutralization prevented the acceleration of lesions in the group where FTY720 treatment was withdrawn at day 10 pi. These mice developed significantly diminished lesions (Figure 4A and B) and angiogenesis (Figure 4C) as compared to group of mice treated similarly with isotype control antibody. Further more anti-IL-6 antibody treated mice developed significantly lower levels of Th17 cells as compared to FTY720 discontinued group in the lymphoid organs when the cells were stimulated in the presence of PMA/Ionomycin (Figure 4E and F). These results indicate that IL-6 cytokine contributes to the lesion rebound.

Further evidence that Th17 cells might be involved in rebound of lesion severity once FTY720 therapy was discontinued came from the experiments performed in IL-17 receptor knockout (IL-17RKO) mice. Ocularly infected IL-17RKO mice developed significantly less severe SK lesions as compared to WT mice as was reported previously [24]. When treatment was withdrawn on day 10 pi. in IL-17KO mice, the rebound in lesion severity was minimal (Figure 4D).

### FTY720 Ligation to S1P Receptors Promotes Differentiation of Th17 as well as Treg Subsets

**Figure 5:**
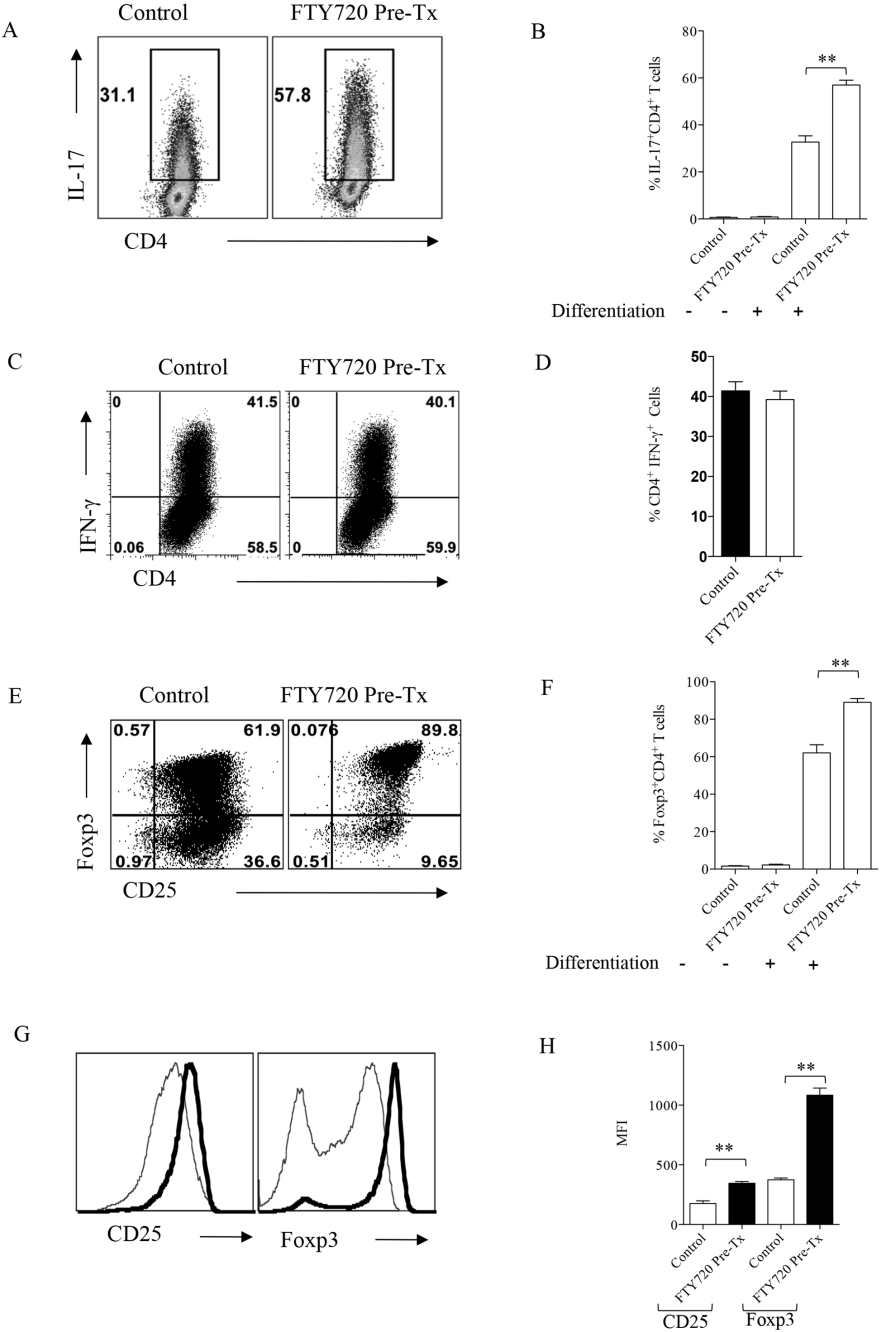
FTY720 ligation to S1P receptors promotes Tregs and Th17 cell differentiation. DO11.10 Rag2−/− mice were treated with 0.3 mg/kg body wt of FTY720 for 7 days. Splenocytes were isolated either from FTY720 treated or control animals and their TCR was stimulated under Th1, Th17 or regulatory T cell polarizing conditions in vitro for 5 days. After 5 days of incubation, cells were stained for measuring the production of cytokines and transcription factor Foxp3 by intra cellular/nuclear staining. (A) FACS plots showing the frequencies of IL-17 producing Th17 cells in FTY720 pre-treated and control groups. (B) Bar graphs for the cumulative data on the frequencies of Th17 cells in two groups. (C) Representative FACS plots showing the frequencies of IFN-γ producing Th1 cells in FTY720 pre -treated and control groups. (D) Bar graphs depict the cumulative data of IFN-γ producing cells from different replicates. (E) FACS plots depicting the frequencies of CD25 and transcription factor, Foxp3 staining in differentiated regulatory T cells in FTY720 pre-treated and control groups. (F) Bar graphs represent the cumulative frequencies of Foxp3^+^ cells in different groups. (G) Histograms show the MFI of CD25 and Foxp3 expression in polarized Tregs in control (faint lines) and FTY720 pre treated (thick lines) groups (H) Bar graphs show the cumulative data of the MFI for CD25 and Foxp3 in control and FTY720 pre-treated group. Data represent means ± SEMs of at least two independent experiments. Statistical significance was calculated by one-way ANOVA with *p≤0.05, **p≤0.01, and ***p≤0.001.

Previous reports have demonstrated that SK is a Th1 dominated immunopathological model [14], [15], however, our data showed that the Th17 cell subtype became prevalent in the FTY720 treatment withdrawal group. Although IL-6, a cytokine essential for Th17 differentiation was found in both FTY720 treated and untreated groups, predominance of Th17 subset over Th1 cells in FTY720 treated animals led us to evaluate the effect of FTY720 on various CD4^+^ T cell subset differentiation. To conclusively address this issue we performed polarization experiments in uninfected DO11.10 Rag2−/− mice. Thus one group of DO11.10 Rag2−/− mice were pretreated with 0.3 mg/kg body weight of FTY720 for 7 days and control group was left untreated. Splenocytes were isolated on day 8 from either FTY720 treated or untreated control animals and stimulated for 5 days *in vitro* with anti CD3/CD28 antibodies under Th1, Th17 or Treg polarizing conditions. After incubation, cells differentiated in Th17 and Th1 conditions were stimulated with PMA/Ionomycin for 4 hrs in the presence of brefeldin A and compared for their production of cytokines by ICCS assay. Compared to untreated controls, CD4^+^ T cells isolated from FTY720 treated animals could be efficiently polarized into cells with Th17 phenotype (Figure 5A and B). However, FTY720 treatment showed no effect on the numbers and frequencies of cells that became differentiated into Th1 phenotype in both the treated and control groups (Figure 5C and D). We could also show that FTY720 treatment resulted in the differentiation of more cells into those with the Treg phenotype than that of control untreated splenocytes (Figure 5E and F). Additionally, the Tregs differentiated from FTY720 treated group expressed higher levels of Foxp3 and CD25 as observed by their mean fluorescence intensities (Figure 5G and H) indicating the critical role played by S1P1 axis in iTreg differentiation. These data demonstrates that CD4^+^ T cells isolated from FTY720 treated mice are sensitized to differentiate both towards Th17 or Treg phenotype rather than the Th1 subtype.

## Discussion

Ocular inflammation induced by HSV infection is an example of an immunoinflammatory lesion with the major orchestrators of the syndrome being proinflammatory CD4^+^ T cells [15], [25]. The extent of tissue damage is limited by several host derived counter inflammatory events one of which is the participation of Foxp3^+^ Treg [3], [26]. Approaches that inhibit effector T cell function or change the balance of T effectors: Tregs benefit the host [2]. The fungal metabolite drug FTY720 can be used to control SK with it acting both to reduce the migration of effector T cells to lesion sites and expanding the representation of Treg [2]. FTY720 has been used to control several inflammatory lesions but lesion relapse can occur if treatment must be discontinued [12]. In the present report, we have evaluated the outcome of treatment discontinuation in the SK model and observed a rapid rebound in the severity of lesions. Moreover, we show that whereas Th17 cells dominated in relapsed SK lesions in untreated animals, the Th1 subset was predominant. A combination of effects appeared to account for these findings. These included more prolonged persistence of virus in animals treated with FTY720, a rapid increase of the cytokine IL-6 upon treatment discontinuation along with the preferential expression of migration molecules on DLN Th17 compared to Th1 cells. The lesion rebound effect was not apparent in animals unable to signal through the IL-17R or in those treated with neutralizing antibody to IL-6. Accordingly, our results demonstrate that when lesion relapses are anticipated control can be effected by forms of therapy that inhibit inflammatory cytokines such as IL-6.

Controlling immunopathological lesions can be achieved in many ways which include the use of FTY720 [5]. The advantage of using drugs such as FTY720 is that it targets two major components of the inflammatory reaction [5], [6], [10]. Thus, inflammatory T cells are sequestered in lymph nodes with their access to tissue sites being obstructed [6], [9], [10]. The drug may also cause a change the balance of functional sets of T cells to one that emphasizes tissue protective Treg responses [5]. When used for sufficient time against SK, FTY720 treatment markedly reduces levels of tissue damage [5]. However, as was noted recently using FTY720 to control an experimental autoimmune lesion, treatment discontinuation can result in a relapse in lesion severity [12], . We found this situation also occurred when treating SK. In fact, the severity of relapsed lesions rapidly became similar in magnitude than those in untreated animals. This observation came as a surprise, but it seemed to be explained by the observation that the cellular drivers of the inflammatory process in relapsed lesion were different and perhaps more potent from those observed in untreated animals. Thus the predominant proinflammatory T cells in relapsed lesions were Th17 cells, the subtype often associated with severe lesions in many autoimmune situations [28], [29], [30], [31]. In untreated animals, Th1 proinflammatory cells dominate in SK lesions, although Th17 cells do become more evident at later stages of SK [24].

Why relapsed lesions were dominated by Th17 T cells remains to be fully explained but several events need to be considered. Firstly, when HSV ocularly infected animals were treated with FTY720, they became less able to control virus and this persisted at least two days beyond what occurred in untreated animals. The persisting virus or the viral antigens could act as a proinflammatory stimulus such as the production of some chemokines and cytokines such as IL-6 which HSV infected cells themselves may produce [22], [23]. The IL-6 cytokine is well known to be a factor involved in the differentiation of Th17 cells from null precursors [30], as was also shown in this report. Also favoring a role for IL-6 in the Th17 dominance was the observation that levels of IL-6 were elevated during relapse and that the rebound lesions could be almost prevented in animals treated with anti-IL-6 neutralizing monoclonal antibody.

Another explanation for our findings is that the Th17 T cells present in relapsed lesions were largely derived from Treg that lost their function in inflammatory environment of the eye or perhaps DLN. Thus, Treg are well known either to lose or take on a proinflammatory function and become mainly Th17 cells when exposed to inflammatory molecules, particularly IL-6 [32], [33]. Moreover, as reported previously one effect of FTY720 treatment is to expand the population of Treg which can be demonstrated both in ocular lesions as well as DLN. It will be of interest to formally show that some of the Th17 cells in the ocular inflammatory population derive from Treg precursors (exTreg). Additionally, it needs to be shown if this proposed Treg plasticity occurs in the DLN and is followed by the migration of exTreg (now Th17) to the eye, or if the plasticity changes occur in the eye itself. It will also be important to determine how the plasticity changes are executed at molecular level and to explore ways to prevent the event from occurring. We are currently attempting to resolve these issues in our laboratory.

Another consequence of FTY720 treatment, we observed was that lymphoid cells taken from uninfected animals treated for some days with FTY720 became more supportive of Th17 and Treg cell induction ex vivo than they were for Th1 cell induction. Although the observation requires a mechanistic explanation, recently it was shown that defects in S1P1 receptor phosphorylation exacerbates Th17 mediated autoimmune neuroinflammation and the increase in Th17 cell population was shown to be dependent on the IL-6-Jak-STAT3 pathway [34]. We are currently measuring if this mechanism is also responsible for the observed tendency of T cells isolated from FTY720 treated DO11.10 Rag2−/− to differentiate preferentially towards Treg and Th17 cells or whether other signaling events are changed by FTY720 treatment such as those mediated by TGF-β.

In conclusion, our data has clarified the conditions under which FTY720 discontinuation leads to recurrence of inflammatory lesions predominated by IL-17 producing Th17 cells. Caution should be taken when FTY720 is used to treat chronic inflammatory diseases where the disease can relapse when treatment is discontinued. During such conditions combination therapy of FTY720 combined with procedures that negate proinflammatory cytokines such as IL-6 may emerge as a broadly applicable approach to treat wider range of inflammatory and autoimmune diseases.

## References

[1] Rowe AM, St Leger AJ, Jeon S, Dhaliwal DK, Knickelbein JE, et al (2013) Herpes keratitis. Prog Retin Eye Res 32: 88–101. 22944008 10.1016/j.preteyeres.2012.08.002 PMC3529813

[2] Veiga-Parga T, Suryawanshi A, Rouse BT (2011) Controlling viral immuno-inflammatory lesions by modulating aryl hydrocarbon receptor signaling. PLoS Pathog 7: e1002427. 22174686 10.1371/journal.ppat.1002427 PMC3234248

[3] J Reddy PB, Schreiber TH, Rajasagi NK, Suryawanshi A, Mulik S, et al (2012) TNFRSF25 agonistic antibody and galectin-9 combination therapy controls herpes simplex virus-induced immunoinflammatory lesions. J Virol 86: 10606–10620. 22811539 10.1128/JVI.01391-12 PMC3457251

[4] Sehrawat S, Suvas S, Sarangi PP, Suryawanshi A, Rouse BT (2008) In vitro-generated antigen-specific CD4+ CD25+ Foxp3+ regulatory T cells control the severity of herpes simplex virus-induced ocular immunoinflammatory lesions. J Virol 82: 6838–6851. 18480441 10.1128/JVI.00697-08 PMC2446952

[5] Sehrawat S, Rouse BT (2008) Anti-inflammatory effects of FTY720 against viral-induced immunopathology: role of drug-induced conversion of T cells to become Foxp3+ regulators. J Immunol 180: 7636–7647. 18490766 10.4049/jimmunol.180.11.7636

[6] Brinkmann V, Billich A, Baumruker T, Heining P, Schmouder R, et al (2010) Fingolimod (FTY720): discovery and development of an oral drug to treat multiple sclerosis. Nat Rev Drug Discov 9: 883–897. 21031003 10.1038/nrd3248

[7] Mandala S, Hajdu R, Bergstrom J, Quackenbush E, Xie J, et al (2002) Alteration of lymphocyte trafficking by sphingosine-1-phosphate receptor agonists. Science 296: 346–349. 11923495 10.1126/science.1070238

[8] Rosen H, Goetzl EJ (2005) Sphingosine 1-phosphate and its receptors: an autocrine and paracrine network. Nat Rev Immunol 5: 560–570. 15999095 10.1038/nri1650

[9] Cohen JA, Barkhof F, Comi G, Hartung HP, Khatri BO, et al (2010) Oral fingolimod or intramuscular interferon for relapsing multiple sclerosis. N Engl J Med 362: 402–415. 20089954 10.1056/NEJMoa0907839

[10] Ingwersen J, Aktas O, Kuery P, Kieseier B, Boyko A, et al (2012) Fingolimod in multiple sclerosis: mechanisms of action and clinical efficacy. Clin Immunol 142: 15–24. 21669553 10.1016/j.clim.2011.05.005

[11] Liu G, Yang K, Burns S, Shrestha S, Chi H (2010) The S1P(1)-mTOR axis directs the reciprocal differentiation of T(H)1 and T(reg) cells. Nat Immunol 11: 1047–1056. 20852647 10.1038/ni.1939 PMC2958252

[12] Yoshida Y, Tsuji T, Fujita T, Kohno T (2011) Relapse of experimental autoimmune encephalomyelitis after discontinuation of FTY720 (Fingolimod) treatment, but not after combination of FTY720 and pathogenic autoantigen. Biol Pharm Bull 34: 933–936. 21628899 10.1248/bpb.34.933

[13] Zhao ZS, Granucci F, Yeh L, Schaffer PA, Cantor H (1998) Molecular mimicry by herpes simplex virus-type 1: autoimmune disease after viral infection. Science 279: 1344–1347. 9478893 10.1126/science.279.5355.1344

[14] Hendricks RL, Tumpey TM, Finnegan A (1992) IFN-gamma and IL-2 are protective in the skin but pathologic in the corneas of HSV-1-infected mice. J Immunol 149: 3023–3028. 1401927

[15] Niemialtowski MG, Rouse BT (1992) Predominance of Th1 cells in ocular tissues during herpetic stromal keratitis. J Immunol 149: 3035–3039. 1357034

[16] Harrington LE, Hatton RD, Mangan PR, Turner H, Murphy TL, et al (2005) Interleukin 17-producing CD4+ effector T cells develop via a lineage distinct from the T helper type 1 and 2 lineages. Nat Immunol 6: 1123–1132. 16200070 10.1038/ni1254

[17] Veldhoen M, Hocking RJ, Atkins CJ, Locksley RM, Stockinger B (2006) TGFbeta in the context of an inflammatory cytokine milieu supports de novo differentiation of IL-17-producing T cells. Immunity 24: 179–189. 16473830 10.1016/j.immuni.2006.01.001

[18] Rajasagi NK, Reddy PB, Suryawanshi A, Mulik S, Gjorstrup P, et al (2011) Controlling herpes simplex virus-induced ocular inflammatory lesions with the lipid-derived mediator resolvin E1. J Immunol 186: 1735–1746. 21187448 10.4049/jimmunol.1003456 PMC3888773

[19] Bettelli E, Carrier Y, Gao W, Korn T, Strom TB, et al (2006) Reciprocal developmental pathways for the generation of pathogenic effector TH17 and regulatory T cells. Nature 441: 235–238. 16648838 10.1038/nature04753

[20] Fujimoto M, Serada S, Mihara M, Uchiyama Y, Yoshida H, et al (2008) Interleukin-6 blockade suppresses autoimmune arthritis in mice by the inhibition of inflammatory Th17 responses. Arthritis Rheum 58: 3710–3719. 19035481 10.1002/art.24126

[21] Halford WP, Gebhardt BM, Carr DJ (1996) Persistent cytokine expression in trigeminal ganglion latently infected with herpes simplex virus type 1. J Immunol 157: 3542–3549. 8871654

[22] Hayashi K, Hooper LC, Chin MS, Nagineni CN, Detrick B, et al (2006) Herpes simplex virus 1 (HSV-1) DNA and immune complex (HSV-1-human IgG) elicit vigorous interleukin 6 release from infected corneal cells via Toll-like receptors. J Gen Virol 87: 2161–2169. 16847112 10.1099/vir.0.81772-0

[23] Kanangat S, Babu JS, Knipe DM, Rouse BT (1996) HSV-1-mediated modulation of cytokine gene expression in a permissive cell line: selective upregulation of IL-6 gene expression. Virology 219: 295–300. 8623544 10.1006/viro.1996.0250

[24] Suryawanshi A, Veiga-Parga T, Rajasagi NK, Reddy PB, Sehrawat S, et al (2011) Role of IL-17 and Th17 cells in herpes simplex virus-induced corneal immunopathology. J Immunol 187: 1919–1930. 21765013 10.4049/jimmunol.1100736 PMC3150378

[25] Niemialtowski MG, Rouse BT (1992) Phenotypic and functional studies on ocular T cells during herpetic infections of the eye. J Immunol 148: 1864–1870. 1347309

[26] Rouse BT, Sehrawat S (2010) Immunity and immunopathology to viruses: what decides the outcome? Nat Rev Immunol 10: 514–526. 20577268 10.1038/nri2802 PMC3899649

[27] Penaranda C, Tang Q, Ruddle NH, Bluestone JA (2010) Prevention of diabetes by FTY720-mediated stabilization of peri-islet tertiary lymphoid organs. Diabetes 59: 1461–1468. 20299465 10.2337/db09-1129 PMC2874707

[28] Tzartos JS, Friese MA, Craner MJ, Palace J, Newcombe J, et al (2008) Interleukin-17 production in central nervous system-infiltrating T cells and glial cells is associated with active disease in multiple sclerosis. Am J Pathol 172: 146–155. 18156204 10.2353/ajpath.2008.070690 PMC2189615

[29] Kolls JK, Lindén A (2004) Interleukin-17 family members and inflammation. Immunity 21: 467–476. 15485625 10.1016/j.immuni.2004.08.018

[30] Bettelli E, Korn T, Oukka M, Kuchroo VK (2008) Induction and effector functions of T(H)17 cells. Nature 453: 1051–1057. 18563156 10.1038/nature07036 PMC6280661

[31] Kebir H, Kreymborg K, Ifergan I, Dodelet-Devillers A, Cayrol R, et al (2007) Human TH17 lymphocytes promote blood-brain barrier disruption and central nervous system inflammation. Nat Med 13: 1173–1175. 17828272 10.1038/nm1651 PMC5114125

[32] Komatsu N, Okamoto K, Sawa S, Nakashima T, Oh-Hora M, et al (2014) Pathogenic conversion of Foxp3(+) T cells into TH17 cells in autoimmune arthritis. Nat Med 20: 62–68. 24362934 10.1038/nm.3432

[33] van Loosdregt J, Fleskens V, Fu J, Brenkman AB, Bekker CP, et al (2013) Stabilization of the transcription factor Foxp3 by the deubiquitinase USP7 increases Treg-cell-suppressive capacity. Immunity 39: 259–271. 23973222 10.1016/j.immuni.2013.05.018 PMC4133784

[34] Garris CS, Wu L, Acharya S, Arac A, Blaho VA, et al (2013) Defective sphingosine 1-phosphate receptor 1 (S1P1) phosphorylation exacerbates TH17-mediated autoimmune neuroinflammation. Nat Immunol 14: 1166–1172. 24076635 10.1038/ni.2730 PMC4014310

